# COVID-19 vaccines and vaccination: how prepared is Africa?

**DOI:** 10.11604/pamj.2021.39.107.27912

**Published:** 2021-06-04

**Authors:** Olumuyiwa Elijah Ariyo, Elijah Kolawole Oladipo, Oluwadamilola Gideon Osasona, Olumide Obe, Folakemi Olomojobi

**Affiliations:** 1 Department of Medicine, Infectious Diseases and Tropical Medicine Unit, Federal Teaching Hospital Ido-Ekiti, Ekiti State, Nigeria,; 2 Department of Microbiology, Laboratory of Molecular Biology, Bioinformatics and Immunology, Adeleke University, Ede, Osun State, Nigeria,; 3 Genomics Unit, Helix Biogen Institute, Ogbomoso, Oyo State, Nigeria,; 4 African Centre of Excellence for Genomics of Infectious Diseases, Redeemers University, Ede, Osun State, Nigeria,; 5 Department of Biological Sciences, Redeemers University, Ede, Osun State, Nigeria,; 6 Ekiti State Ministry of Health and Human Services, Ado-Ekiti, Ekiti State, Nigeria

**Keywords:** COVID-19, vaccines, vaccine hesitancy, Africa, vaccine nationalism

## Abstract

The approval of vaccines for emergency use signifies a great milestone to end the COVID-19 pandemic. However, less than 2% of the global vaccines have been administered in Africa, putting the continent in a precarious situation in the eventuality of another wave that may consume its health system. There is still an enormous task in Africa in the face of vaccine nationalism. In most countries, vaccine acquisition and deployment have been suboptimal. Leaving out Africa in the race to achieve global herd immunity may be catastrophic. Stakeholders must continue engagement to ensure a successful deployment of the vaccines on the continent. There is a need to build capacity in Africa for rapid vaccine development and deployment in the long term.

## Commentary

The approval of COVID-19 vaccines for emergency use is a great milestone in the battle against the ongoing pandemic, up to 8 vaccines including Pfizer-BioNTech, Moderna, Oxford-Astrazeneca, and most recently Sinopharm has been approved. The vaccine deployment was a turning point in the battle against the COVID-19 pandemic. However, this has mainly been successful in high-income countries [1]. Although this may signal the beginning of the end for the COVID-19 pandemic, nevertheless there is still an enormous task ahead, especially in Africa. With its myriad of challenges, Africa is being left out, and strategic, timely, and systematic interventions need to be instituted to catch up with other parts of the globe. Foreseeably, vaccine deployment is being hampered by issues bordering on the acquisition, handling, storage, distribution, penetrance, and acceptance.

It appears the survival of the fittest is playing out with the COVID-19 pandemic, with many countries, especially in the West, securing sufficient doses of COVID-19 vaccines for their population. However, the situation is still largely bleak in many African countries. Solely responsible for this significant problem of access is the potential inability to source or mobilize adequate funds to acquire and distribute the COVID-19 vaccines. The COVAX facility co-led by the World Health Organisation (WHO), GAVI, the Vaccine Alliance, and the Coalition for Epidemic Preparedness Innovations (CEPI) in partnership with other stakeholders, has made significant efforts towards access to COVID-19 vaccines for Africa, in addition to the African Union´s African Vaccine Acquisition Task Team and bilateral agreement of some countries with vaccines manufacturers. Despite this, less than 2% of about 700 million doses of COVID-19 vaccines administered globally have been in Africa, with 10 countries accounting for 65% of total doses out of the 45 nations that have taken delivery of the vaccines [2, 3]. Africa`s population is 17% of the world population but it has administered the least doses of COVID-19 vaccines. It is essential to highlight the likelihood of further plunging the continent into debt and impoverishment in case of any loans for the vaccine´s procurement. According to the African Centre for Diseases Control (CDC) Director, a delay in attaining wide coverage of the COVID-19 vaccine in Africa may be catastrophic [4]. Despite the uncertainties and disadvantaged position among the comity of nations, there are projections that 20% or 600 million doses of the vaccines will be available to African dwellers by the end of 2021 [2]. This, however, raises the issue of sustainability of vaccine availability and how long it will take Africa to vaccinate the critical mass of its population to attain herd immunity. This put the continent in a precarious situation as another wave may be far more devastating than what is currently being reported in India, considering mutant strains emergence [5]. Unarguably, equitable access to vaccines around the world would be key, in making success, in the fight against COVID-19.

Furthermore, the readiness of some African countries to take delivery and distribute the vaccines is still not optimal. A World Health Organisation (WHO) report showed that the Africa region had 33% readiness for COVID-19 vaccine roll-out [6]. Critical components for the roll-out include infrastructure, logistics, human resources, and vaccine deployment surveillance, among others. The crucial place of maintaining the vaccine in the cold chain cannot be overemphasized. For instance, Pfizer-BioNTech requires storage below - 70°C, which may not be feasible for most African countries due to insufficient power infrastructure. Hence, there may be a need to invest in sustainable innovations that can solve the storage debacle to roll it out successfully. In addition to this, logistic impediments may preclude access to the remote, underserved, and difficult to reach populations. The training and capacity building for vaccination personnel and continuous surveillance for vaccine safety are also important. For the latter, information management, in respect of adverse events following immunization, is a delicate balance, as it could heighten suspicion and impact vaccine acceptance negatively [7]. The report of a serious adverse event like blood clots was seen with Johnson and Johnson and AstraZeneca, and this has made some countries limit their use to those above 50-60 years; with Africa's young population and COVAX facility delivery of mainly Astrazeneca vaccines, such may not be easy to implement in Africa, but it is crucial to continuously assess the situation in addition to the reassurance given by regulatory bodies in Europe and other parts of the world.

The scale of priority on the administration of the vaccines is very important. A salient question, at this point, will be the length of time it would take for Africa to vaccinate a critical number of its population. A minimum of 60% or almost 800 million will need to be vaccinated to achieve this. In most of the countries where the vaccines have been deployed, the priority was to frontline healthcare workers and the vulnerable. In Africa, there is a need to address existing health equity issues so that the socially and medically vulnerable populations will have access to the vaccines on time. Another factor that is critical to the success of the vaccine is acceptability. Apart from diverse forms of misinformation and disinformation, another prevalent characteristic in many African societies is “COVID-19 doubters”. Like the Human Immunodeficiency Virus (HIV) experience, many people vehemently deny the existence of the virus. Many of the enlightened population expound on several conspiracy theories, both about the disease and the vaccine; thus, impacting negatively on risk perception, compliance, and readiness to accept vaccination. A study showed that about 20% of Nigerians are unwilling to take the COVID-19 vaccines with another 22% indecisiveness [8]. This may be much more in reality. Myths and misconceptions such as the ability of the vaccines to alter DNA, cause infertility, or its use to control population are some of the reasons that could prevent its uptake. Therefore, it is essential to tackle myths and misinformation about COVID-19 and vaccine hesitancy to ensure an adequate number of the population is vaccinated. The efficacy and effectiveness of the COVID-19 vaccines among the African population require a continuous evaluation besides rigorous safety assessment. There is a need for National Regulatory Authorities (NRA) in Africa to make available a clear ready-made pathway on the approach to conducting a rapid assessment that will determine the use or otherwise of any COVID-19 vaccine that has been given emergency approval. A joint regulatory review by African NRAs may be a way out in collaboration with the WHO. Also, active pharmacovigilance will be crucial in monitoring the safety profile, including the vaccine's long-term effects.

In December 2020, the Africa CDC, in conjunction with Africa Union and South Africa Medical Research Council, held a virtual conference on a framework for fair, equitable, and timely allocation of COVID-19 vaccines in Africa [9]. This was followed in April 2021 by another stakeholder’s conference with an ambitious target of the continent producing 60% of the vaccines needed by 2040 [10]. These are laudable steps that show the readiness and alertness of Africans to play a part in achieving the requisite targets. It is important to sustain stakeholder interactions across the different geographical and professional divides along these lines. The roles of the private sector, as well as the ethnocultural and religious institutions, cannot be overemphasized, as all these have specific and far-reaching roles to play across the facets of ensuring the successful distribution of COVID-19 vaccines in Africa. In the long term, there is a need for Africa to build capacity in all aspects of vaccine development and deployment from “the genes to the globe” and reduce her reliance on external supports to cater to her people. The time for Africa to be ready is now.

## References

[1] The New York Times Covid-19 Vaccine Tracker.

[2] WHO COVID-19 vaccines. Regional Office for Africa.

[3] WHO Less than 2% of the world´s COVID-19 vaccines administered in Africa. Regional Office for Africa.

[4] Global news Delayed coronavirus vaccine in Africa risks´ moral catastrophe, CDC chief says - National. Globalnews.ca.

[5] The Guardian Covid third wave may overrun Africa´s healthcare, warns WHO | Global development.

[6] WHO WHO urges African countries to ramp up readiness for COVID-19 vaccination drive. Regional Office for Africa.

[7] WHO COVID-19 vaccine country readiness and delivery.

[8] Olomofe CO, Soyemi KV, Udomah BF, Owolabi AO, Ajumuka EE, Igbokwe MC (2021). Predictors of Uptake of a Potential Covid-19 Vaccine Among Nigerian Adults. J Vaccines Vaccin.

[9] Africa CDC Virtual Conference: Framework for Fair, Equitable and Timely Allocation of COVID-19 Vaccines in Africa.

[10] Africa CDC African Union and Africa CDC launches Partnerships for African Vaccine Manufacturing (PAVM), the framework to achieve it and signs 2 MoUs.

